# An approach to incorporate individual personality in modeling fish dispersal across in‐stream barriers

**DOI:** 10.1002/ece3.2629

**Published:** 2016-12-23

**Authors:** Philipp Emanuel Hirsch, Magnus Thorlacius, Tomas Brodin, Patricia Burkhardt‐Holm

**Affiliations:** ^1^ Research Centre for Sustainable Energy and Water Supply; ^2^ Program Man‐Society‐EnvironmentDepartment of Environmental SciencesUniversity of BaselBaselSwitzerland; ^3^ Department of Ecology and Environmental ScienceUmeå UniversityUmeåSweden; ^4^ Department of Biological SciencesUniversity of AlbertaEdmontonABCanada

**Keywords:** behavioral syndromes, ecological modeling, habitat fragmentation, invasive species, propagule pressure

## Abstract

Animal personalities are an important factor that affects the dispersal of animals. In the context of aquatic species, dispersal modeling needs to consider that most freshwater ecosystems are highly fragmented by barriers reducing longitudinal connectivity. Previous research has incorporated such barriers into dispersal models under the neutral assumption that all migrating animals attempt to ascend at all times. Modeling dispersal of animals that do not perform trophic or reproductive migrations will be more realistic if it includes assumptions of which individuals attempt to overcome a barrier. We aimed to introduce personality into predictive modeling of whether a nonmigratory invasive freshwater fish (the round goby, *Neogobius melanostomus*) will disperse across an in‐stream barrier. To that end, we experimentally assayed the personalities of 259 individuals from invasion fronts and established round goby populations. Based on the population differences in boldness, asociability, and activity, we defined a priori thresholds with bolder, more asocial, and more active individuals having a higher likelihood of ascent. We then combined the personality thresholds with swimming speed data from the literature and in situ measurements of flow velocities in the barrier. The resulting binary logistic regression model revealed probabilities of crossing a barrier which depended not only on water flow and fish swimming speed but also on animal personalities. We conclude that risk assessment through predictive dispersal modeling across fragmented landscapes can be advanced by including personality traits as parameters. The inclusion of behavior into modeling the spread of invasive species can help to improve the accuracy of risk assessments.

## 1 Introduction

### 1.1 Animal personality is important especially in invasive species management

Animal personality fundamentally affects the space use of individuals (Conrad, Weinersmith, Brodin, Saltz, & Sih, 2011). For example, personality‐dependent movement patterns of individuals ultimately predict how a population moves through the environment (Harrison et al., 2014). There is growing evidence that individual personality is related to individual dispersal tendency (Cote, Clobert, Brodin, Fogarty, & Sih, 2010). Personality‐dependent dispersal might be an especially important factor determining the establishment success of animal invasions. Modern modeling frameworks and experiments suggest that incorporating animal personalities into invasion models helps better explain invasion success. Recent theoretical work suggests that the presence of bolder, more asocial, and more active individuals increases the probability that an invasive population spreads further (Chapple, Simmonds, & Wong, 2012). The underlying rationale is that asocial and bold individuals will colonize empty landscape patches sooner, thus inducing a faster dispersal and population growth at the invasion front (Fogarty, Cote, & Sih, 2011). Our study aims to underpin this theoretical framework by incorporating assumptions based on experimentally tested animal personalities into modeling the likelihood of dispersal across barriers. The overarching goal is to present an approach on how to make models and ultimately risk assessments on invasive species dispersal across barriers more realistic.

### 1.2 Dispersal over barriers by freshwater fish and invasive species management

The dispersal of animals through pristine landscapes, which are devoid of man‐made dispersal barriers can be modeled with increasing accuracy. For example, the spread of introduced brown trout (*Salmo trutta*) into pristine river systems in Greenland could be modeled and predicted with high accuracy (Labonne et al., 2013). However, most ecosystems today are highly fragmented which may affect the predictive power of dispersal models. Research on the dispersal of terrestrial species has long acknowledged that roads pose effective migration barriers (Shepard, Kuhns, Dreslik, & Phillips, 2008). River systems are even more drastically affected by fragmentation as artificial structures such as dams or culverts drastically reduce a river's longitudinal connectivity (Crook et al., 2015). A globally relevant threat to biodiversity are species invasions. The dispersal of invasive freshwater fish is a particular threat to native freshwater biota on a global scale (Gozlan, Britton, Cowx, & Copp, 2010). Consequently, there is a growing interest in exploring whether and how existing structures such as hydropower dams might impede or even stop the natural dispersal of invasive species (Frings et al., 2013; Hermoso, Januchowski‐Hartley, & Linke, 2015). Assessing the risk of whether an invasive species’ spread will be impeded by a reduced longitudinal connectivity is an essential component of the precautionary approach to species invasion management (Leung et al., 2002). Such an assessment inevitably happens in a situation of incomplete knowledge because the invasive species is still absent and risk assessments must rely on predictive approaches. Predictive dispersal modeling can generate scenarios on which conservation managers and decision makers can base their prioritization of preventive actions (N'Guyen, Hirsch, Adrian‐Kalchhauser, & Burkhardt‐Holm, 2015). Here, we aim to advance the realism of such predictive modeling by incorporating animal personality as a previously underappreciated factor in determining the spread across barriers.

### 1.3 Incorporating personality into modeling to improve risk assessment for management

Most commonly, a modeling approach is used to assess the ecological risk (or benefit) of a barrier blocking longitudinal connectivity (Crook et al., 2015). For example, based on swimming ability and water discharge through a fish ladder, researchers can create a hypothetical probability of passage of a fish across the barrier (Starrs, Ebner, Lintermans, & Fulton, 2011). However, such models are traditionally computed under the neutral assumption that all fish would actually attempt to overcome the barrier. Even in situ tests of fish passage in in‐stream barriers rarely appreciate individual differences in the propensity to ascend. Fish are typically captured, tagged, and displaced downstream, with the implicit assumption that all individuals would display equal and full propensity to ascend (Forty, Spees, & Lucas, 2016). If it is assumed that all fish try to ascend at all times, there are only two limits to success of ascent: the fish swimming performance and the flow velocity within the fish ladder (Radinger & Wolter, 2014; Starrs et al., 2011). Applying such simple limits to species that show trophic or reproductive migrations is reasonable (Brodersen et al., 2011; Northcote, 1997). However, applying the neutral assumption of a “constant intention” to ascend is rather artificial in the case of nonmigratory species. Model assumptions that comprise limits for individual propensities to ascend should represent a more accurate picture of reality. Even if such limits cannot be determined by field observations but must be set a priori, as in the case of predictive modeling, they would account for the dispersal intention of nonmigratory species to ascend. Recent theoretical advances have acknowledged that personality influences an animal's “intention” to disperse (“intention” in sensu Morales et al. (2010); Canestrelli, Bisconti, & Carere (2016)). Therefore, our study seeks to complement the traditional approach of modeling upstream passage by adding personality traits as proxies for the “intention” to ascend.

### 1.4 Case study—round goby and the need for a risk assessment incorporating personality

**Figure 1:**
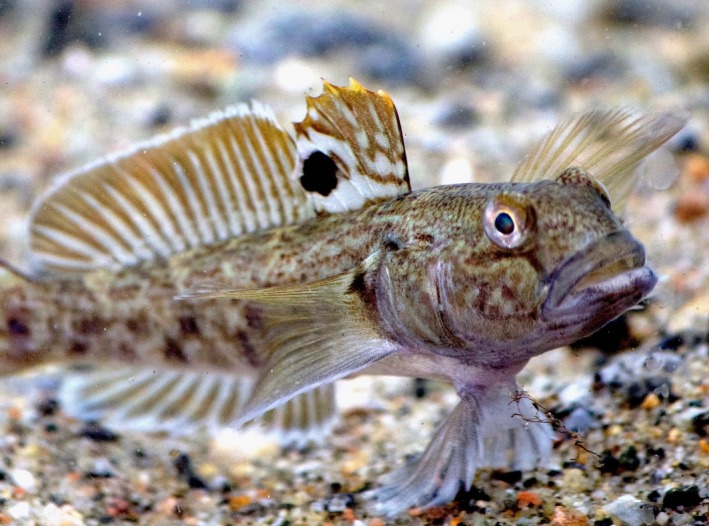
The study species. A round goby (*Neogobius melanostomus*) displaying its characteristic black spot in the first dorsal fin and its fused pelvic fin. Picture: Magnus Thorlacius

**Figure 2:**
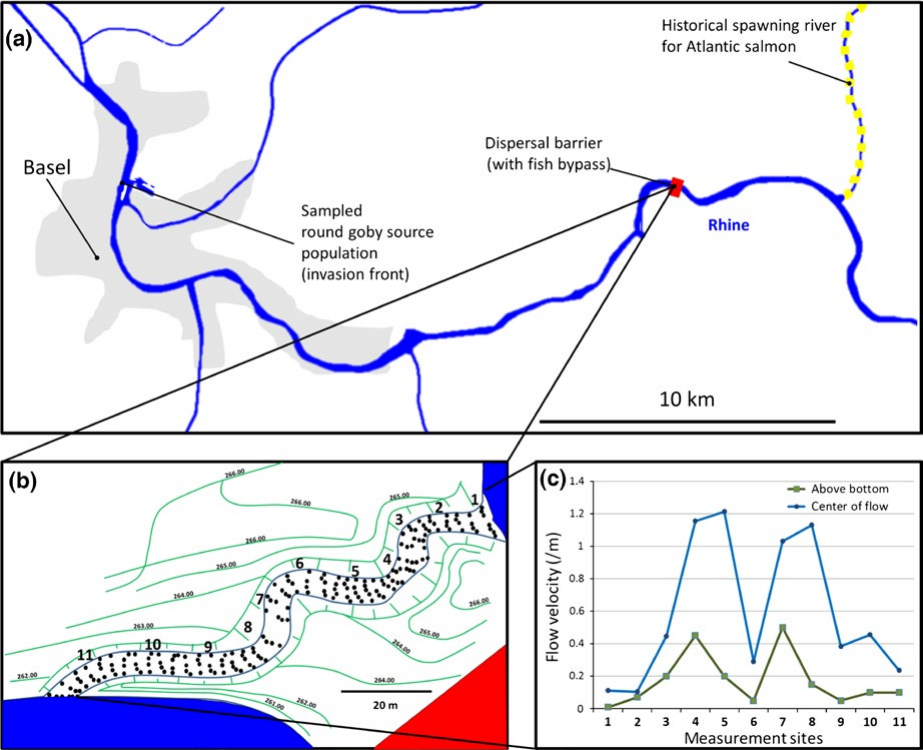
Invasion threats from round goby and specifications of the studied dispersal barrier. (a) Geographical overview of the case study sites including the current source population where round goby were first detected in 2012. The dispersal barrier is a hydropower dam located approx. 5 river kilometer downstream of the river Wehra which is one of the tributaries in this area that historically featured Atlantic salmon (*Salmo salar*) spawning grounds (IKSR, 2009). Further round goby invasion beyond the barrier might compromise undergoing efforts to restore historical salmon populations because round goby might curb salmon reproduction via egg predation (see main text for further explanation). (b) Overview of the fish bypass around the hydropower dam. Green lines indicate topography with elevation in m.a.s.l. Black dots indicate artificially created riffles and spaces in between indicate pools. Numbers 1–11 indicate sites of flow velocity measurements. (c) Flow velocity data from all 11 measurement sites categorized by measuring positions in the water flow as near bottom (=directly above the bottom in the center of the stream) or center of stream (=at 5–30 cm depth in the center of the flow, depending on conditions at the measurement site). Detailed flow velocity profiles for each site are available in Appendix S1

We test our approach by means of a case study: the invasive round goby (*Neogobius melanostomus*) in the River Rhine in Switzerland. The round goby is a small bottom‐living fish native to the Ponto‐Caspian area (Figure 1). In 2012, an invasive round goby population was detected in a harbor of the city of Basel (Kalchhauser, Mutzner, Hirsch, & Burkhardt‐Holm, 2013). This raised concern among scientists since round gobies in other systems have brought substantial changes to those ecosystems (Hirsch, N'Guyen, Adrian‐Kalchhauser, & Burkhardt‐Holm, 2015). Round gobies are known as egg predators of salmonids, and in North America, their establishment has compromised expensive restoration efforts for salmonid spawning grounds (Fitzsimons, Williston, & Williston, 2006; Markham et al., 2008). River restorations in Switzerland are also conducted to allow Atlantic Salmon (*Salmo salar*) to spawn in Rhine tributaries upstream of the current invasion front of round goby in Basel (Figure 2a). Consequently, ecological and economic effects are also predicted if round goby disperse further upstream into the goby‐free river reaches (Verliin et al., 2016). A hydropower dam that was recently refurnished with a fish bypass to facilitate upstream migration of native freshwater fish constitutes a potential barrier to the further upstream spread of the source population in Basel (Figure 2a). Because of the potentially far‐reaching ecological and economic consequences of a round goby range extension, a risk assessment is required. This risk assessment needs to make predictions about how likely it is that round goby ascend across in‐stream barriers upstream of their current distribution. Here, we take a three‐step approach to remedy the current lack of risk‐related information on round goby spread. First, we analyzed the factors posing barriers for ascent in a major in‐stream barrier (flow velocities) (Step 1). Then, we analyzed the personality traits of round gobies in experiments. Based on preexisting knowledge on swimming speeds (compiled in Step 2) and personality‐dependent dispersal, we define a priori thresholds for an individual's “intention” to ascend. We then incorporate in situ measurements and a priori set thresholds to ascend as factors into a predictive model of fish ascent (Step 3).

## 2 Materials and Methods

### 2.1 Predictive risk assessment*—*statistical modeling of ascent

We built on an established approach to model upstream dispersal of fish across barriers that is based on flow velocity and swimming speed. This classical approach was most recently applied by Starrs et al. (2011) who combined swimming speed measures with flow velocity measures to assess the probability that Macquarie perch (*Macquaria australasica*) ascend across culverts. We complemented this approach by incorporating thresholds derived from behavioral data into the modeling. The underlying mathematical model remains unchanged: a generalized nonlinear model of a binary logit regression. The binary response variable is “success” or “failure” of ascent across a migration barrier. The procedure is as follows: First, a decision matrix is created. The matrix contains a series of questions in which an individual's swimming or behavioral trait values are compared with an a priori defined threshold. Surpassing this threshold or not determines success or failure. Next, the relationship between success and failure and the combination of the a priori defined thresholds and the empirically measured predictor variables is explored statistically. The decision matrix was constructed in Excel, all modeling was performed in Statistica v. 11.0, and personality data were analyzed in R.

### 2.2 Step 1

#### 2.2.1 Model parameterization I: flow measurements in the barrier

The bypass of the power plant used in this study was specifically designed to allow fish to migrate upstream. It consists of a flow stretch of approx. 65 m length and 3 m width with a slope of 3.75% (2.2°). It contains 34 riffles and pools created by large stones (Figure 2b). The flow velocity measurements were taken at every third riffle resulting in 11 measurements in 6 m intervals across the 65 m flow distance (Figure 2b,c). Measurements were conducted by monitoring flow velocity with a flowmeter (Schiltknecht) for 30 s at three different locations (above ground, center of flow, and near shore). Measurements were taken the 20th of August 2013 at 18°C water temperature. During the measurements, the Rhine had a water level of 262.750 m.a.s.l. The discharge in the Rhine was typical for this time of the season. Mean temperature averaged across 1971–2014 measured at a close‐by monitoring station were 19°C in June through August (data obtained from the Federal Office for the Environment hydro monitoring station Rheinfelden). Across the year, the bypass has a mean discharge of approx. 0.2/m^3^ with a mean water depth of approx. 0.30 m and a maximum depth of 0.60 m. It should be noted here that the operator of the power plant is legally obliged to provide these levels of water and discharge as a residual flow all year round to allow fish to migrate. Flow velocities are therefore unlikely to be lower at any other day of the year because, legally, the discharge must not decrease. Any increase in discharge will be directed through the turbines by the operator who aims to maximize energy production (Hirsch, Schillinger, Weigt, & Burkhardt‐Holm, 2014; Peter, 2010).

### 2.3 Step 2

#### 2.3.1 Model parameterization II: analyzing size‐dependent swimming speed and endurance time and setting thresholds

Experimentally measured swimming speeds of fish are most commonly used to derive field‐applicable water velocity criteria for fish passages. We therefore conducted a literature survey on swimming speed in gobiid and nongobiid benthic fish. Because body length is decisive for the swimming speed and endurance of fish in general, because it was directly obtainable from all analyzed fish, and because it could covary with personality traits, we included total length (TL) as an explanatory variable into the model.

### 2.4 Step 3

#### 2.4.1 Model parameterization III: analzing fish personality traits and setting thresholds

Fish were caught in the Rhine in Basel, Switzerland (47°35′18″N, 7°35′29″E), in March 2014 using minnow traps and fyke nets. The fish were acclimatized in 80 × 40 × 45 cm aquaria with 5 cm of gravel and PVC pipes as shelters provided in excess. Tap water at temperatures ranging between 10 and 12°C was provided in a flow‐through system, and fish were fed with chironomids until 2 days prior to the shipping to the experimental facilities which took approx. 20 hr. Mortality during shipping was <5%, and upon arrival, the fish were acclimatized to large basins (110 × 110 × 100 cm) with a light:dark cycle of 14:10 hr, 10–14°C tap water supplied as flow through and also fed chironomids daily. After 5 days of acclimatization, they were anaesthetised with MS‐222, measured for TL to the nearest 0.1 cm and individually marked with glass‐encapsuled (8 × 1.4 mm) Biomark^®^ PIT‐tags. Mortality after tagging was <3%. Sizes ranged from 7.3 to 15.3 cm TL with a mean of 9.9 cm. One week after marking, assays of boldness, sociability, and activity were conducted with 50 individuals randomly chosen from the tank. Prior to all behavioral assays, each individual's tag was recorded and fish were allowed to acclimatize individually in 10‐L containers prior to all assays.

##### 2.4.1.1 Boldness

All behavioral assays followed previously described standard procedures and were recorded with Logitech web‐cameras and subsequently analyzed using the open‐source software iSpy (iSpyconnect.com) (Thorlacius, Hellstrom, & Brodin, 2015). We used the most commonly applied standard measure of boldness in fish which is risk tolerance or latency to regain normal behavior after a risky encounter. At many fish ladders, predatory birds await ascending fish to prey on them. In the River Rhine near the fish bypass, several fish‐eating predatory birds can be observed. Especially, cormorants (*Phalacrocorax carbo*), which are known to prey heavily on round goby (Somers, Lozer, Kjoss, & Quinn, 2003), are frequently seen (P.E. Hirsch, personal observations). Based on this information, an artificial bird beak was constructed using a small PVC pipe and dark gray hard plastic sheet sawed into the shape of a beak. Each individual goby was isolated in a 10‐L container for 1 hr to standardize handling and stress levels before being placed in the experimental aquarium (60 L, 80 × 26.5 × 30 cm with nontransparent sides and a camera above). In the aquarium, each individual was left to acclimate for 10 min before the recording started and the beak was released such that it penetrated the water surface approximately one body length away from each fish. The beak was then immediately retracted. Round goby responded in one of two ways to the simulated attack: Either they would freeze immediately (immobility), or they would swim forcefully for a few seconds (escape response) before freezing. Boldness was recorded from the videos as latency to first movement (seconds) following freezing. After each trial, the fish were returned to their individual 10‐L container for 1 hr before the sociability assay started. 75% of the water in the boldness aquarium was replaced between trials in order to reduce chemical cues carrying over between individuals/trials. To ensure that higher values represent higher boldness (less freezing time), we transformed boldness values by log(3600)‐log(time to move) where 3600 is the maximum time, in seconds, for the behavioral assay.

##### 2.4.1.2 Asociability

Asociability was measured in 60‐L aquaria (80 × 26.5 × 30 cm) that were divided into three compartments using transparent hard plastic. The middle compartment comprised half of the volume and each end compartment one‐fourth. Every day, 1 hr before beginning the experiments, two medium‐sized round gobies were placed in one end compartment and the other was left empty. The focal individual was placed in the middle compartment and recorded without disturbance for 1 hr. Individuals that did not move during the first 30 min of the video were excluded from the analysis because asociability could not be recorded for a sufficient amount of time. Here, 75% of the water was also replaced between trials. One frame per 6‐s was later extracted from each recording, using the open‐source software ffmpeg (ffmpeg.org), starting when the fish began to swim. Extracted frames were then used to analyze the distance of the assayed fish from the stimuli pair. This was performed by extracting *x*–*y* coordinates (measured in pixels using the open‐source software ImageJ (imagej.net)) by selecting one point between the eyes of the focal individual in each frame from which average distance to the stimuli pair could be estimated and converted to cm using the width of the aquarium as a reference. Deviating from the convention to label this personality trait sociability, we used the term asociability because this more specifically describes how increasing numerical values of the index describe less social individuals.

##### 2.4.1.3 Activity

Activity was calculated from the asociability data as the sum of moves longer than one centimeter per 6‐s (extracted frame). A high score indicates high activity.

##### 2.4.1.4 Above‐average boldness, asociability, and activity as thresholds for success

To include animal personality as an added explanatory variable into the decision matrix and thus into the model, we had to a priori set thresholds that determine success or failure. To base these a priori thresholds for when an individual has the “intention” to ascend on empirical data, we compiled our own published and unpublished data on personality differences between “invasion center” established and “invasion front” dispersing populations of round goby. Empirical work in round goby found individuals from the invasion front to be bolder and to move faster and further in an experimental stream (Myles‐Gonzalez, Burness, Yavno, Rooke, & Fox, 2015; Thorlacius et al., 2015). Similarly, round gobies in newly colonized areas were bolder, more asocial and active than conspecifics from older populations (Thorlacius M., Hellström G., Finn F., Boman N., & Brodin T, unpubished data), and dispersal tendency has been correlated with boldness (Cote, Fogarty, Brodin, Weinersmith, & Sih, 2011), asociability (Cote et al., 2010), and activity (Thorlacius et al., 2015) in invasive fish. Boldness and activity, which are often correlated (Riechert & Hedrick, 1993), have been associated with foraging and mating success as bold and active individuals spend more time exploring their surroundings and are thereby more likely to find food and mating opportunities (Dingemanse et al., 2007; Sih, Kats, & Maurer, 2003). Activity has also been found to be positively correlated with velocity in ascending a fish ladder in the silver redhorse (*Moxostoma anisurum*) (Silva et al., 2015). Asocial individuals are typically more prone to disperse because they would leave an existing group to explore an environment possibly not containing any congeners (Cote, Dreiss, & Clobert, 2008). There are, of course, exceptions to these rules but, based on existing data, we deemed such a generalization applicable. The rationale for thresholds was that the mean values of personality traits found in individuals sampled at newly dispersed “invasion front” sites are what makes for “good dispersers” whereas lower‐than‐average values are indicative of more resident individuals as found in established invasion centers.

**Figure 3:**
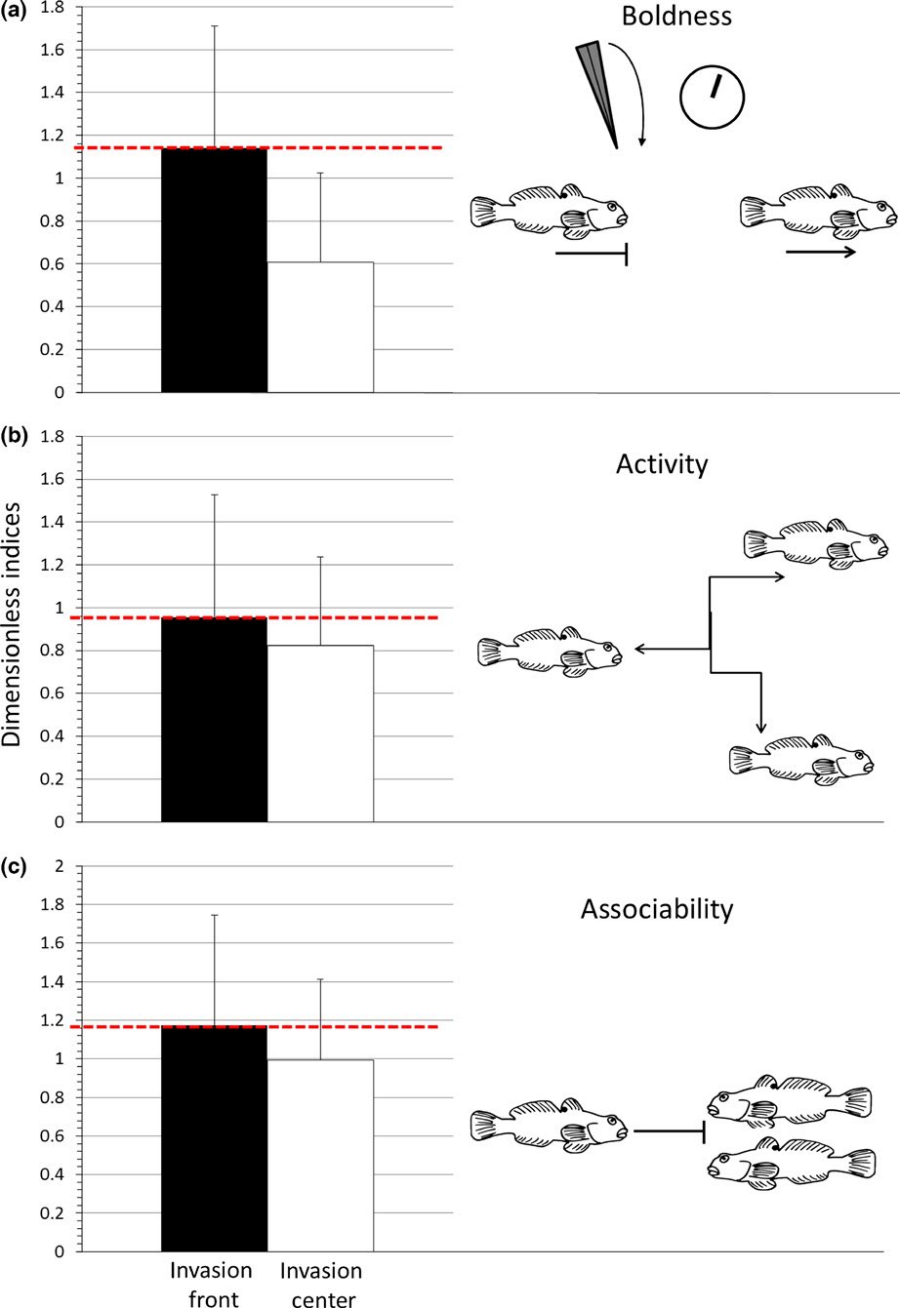
Personality traits differ across invasion stage. Comparison of boldness (a), activity (b), and asociability (c), of round goby individuals sampled from three sites at “invasion fronts” (*n *=* *161) and from two “invasion centers” (*n *=* *98). All data are from own analyses. Indices of personality traits are presented as dimensionless for brevity, please refer to Section “2” for details. Data include traits of individuals analyzed for the source population studied here (*n *=* *50, “invasion front” population from Basel, Switzerland). For more details on the differences between invasion front and center see the main text. The red‐dotted line indicates the threshold for boldness that we applied for our decision matrix

Invasion center and invasion front sites were selected based on published data and fishing records for well‐studied round goby populations in Scandinavia and in Switzerland. The compiled data were all analyzed by us, based on standard procedures (Thorlacius et al., 2015). Individuals were sampled from two “invasion center” sites (*n* = 98) from the Gulf of Gdansk (54°10′52″N, 18°32′23″E, Poland) (Sapota & Skóra, 2005) and three “invasion front” sites (*n* = 161) from Åland (60°06′01″N, 19°55′23″E, Finland), Gotland (57°38′17″N, 18°17′13″E, Sweden), and Basel (Switzerland, see above). Published records indicate an age of the “invasion centers” of approximately 15 years (Sapota & Skóra, 2005). Invasion fronts were approximately 1–2 years old (Kalchhauser et al., 2013; Thorlacius et al., 2015) (Figure 3). Sizes of fish did not differ significantly between invasion front and center (Appendix S2). The data from different populations were acquired using comparable methodologies except for the sociability trials which differed in total duration. To account for this difference in methodology, the activity recordings were expressed as percentage of total observations (see Appendix S3 for details). We also confirmed that the arithmetic mean truly represents a population mean by exploring the skewness and kurtosis of all three measured traits for the fish from Basel (Appendix S4). If an individual had higher values than the mean, we decided that it ascended (decision = 1). We therefore built the decision matrix so that if an individual obtained a 1 at two of the three values we decided it ascended. We deemed that the three traits boldness, activity, and asociability should allow compensating for each other, at least to some degree. In the context of dispersal, it has been shown that all three traits increase the propensity to disperse in mosquitofish (*Gambusia affinis*) (Cote et al., 2011). Increased boldness and activity have also been found to be correlated and to increase dispersal tendency in artificial dispersal systems (Thorlacius et al., 2015). Field data from dispersing round goby individuals from invasion fronts relative to longer established populations also confirmed that dispersing individuals’ traits of activity and boldness are correlated (Myles‐Gonzalez et al., 2015). Whether or not boldness is a positive trait in terms of increasing survival or fitness is most likely a context dependent and complex question (Jolles, Manica, & Boogert, 2016). However, the scientific knowledge regarding the importance of boldness for dispersal in round goby is solid. Based on this knowledge, we created a compensatory threshold: If, for instance, an individual is above‐average bold, above‐average asocial but below‐average active, we still assigned a 1 (for success) for its intention to ascend. If an individual had a trait value lower than the mean in two traits, we decided that it did not have the intention to ascend.

## 3 Results

### 3.1 Step 1. Flow measurements in the barrier

Flow velocities near the shoreline were much lower than in the center. Highest velocities were recorded at 15 cm depth near the shoreline (0.16 m/s) and in the center (1.4/m) (Figure 2, Appendix S1). Flow velocities above the bottom in the middle of the flow were comparable across stations and much lower than closer to the water surface with a maximum of 0.52 m/s. Because the round goby lacks a swim bladder and rarely swims into the water column, we deemed the bottom values as a realistic threshold. It should be noted that near‐bottom flows close to the shoreline were substantially lower than flow in the middle of the bypass (Figure 2).

### 3.2 Step 2. Analysis of size‐dependent swimming speed and endurance time

**Figure 4:**
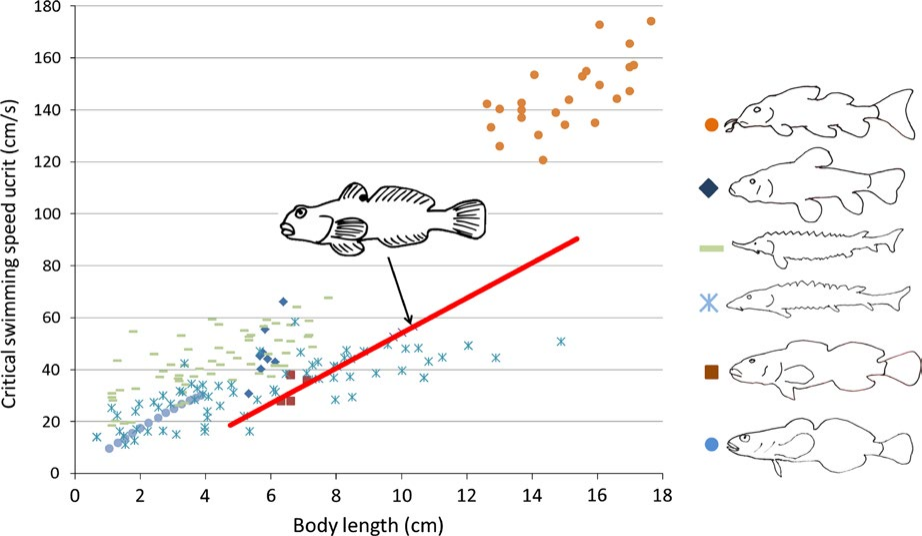
Published data suggest that swimming speed increases linearly with body length. Relationship between body length and swimming speed as extrapolated from published data for round goby (red regression line, graphical depiction showing a round goby) and other species similar to round goby in size and benthic lifestyle. Nongoby species from top: *Leporinus reinhardti, Erimyzon sucetta, Acipenser medirostris, Acipenser transmontanus, Cottus gobio, Cottus bairdi*. See Appendix S5 for details on data sources and species

The literature survey revealed six published studies on swimming parameters of round gobies in both native and invasive populations. Instead of extracting data for only the limited size ranges applied in all studies, we used individuals’ body size (measured as TL) as a proxy for swimming performance. The most commonly applied measure was critical swimming speed (Ucrit), a measure of maximum sustained speed. It is generally assumed that Ucrit allows for conservative estimates of maximum water velocity against which a fish can still swim (Peake, 2008). Swimming capacity of fish typically increases linearly with size because the energy stores needed to support burst activity scale with body size (Kieffer, 2000). When fish approach their maximum size, other parameters limit swimming performance (Martínez et al., 2003), but a general increase in swimming performance with body size can be assumed between juvenile and adult sizes of a species (Kieffer, 2000). For verification of this relationship, we compiled data for body size and swimming speed for a variety of fish species that are similar to round goby (Figure 4). We mined the following papers to compile a relationship between fish size, swimming speed, and endurance of the round goby (Hoover, Adams, & Killgore, 2003; Myles‐Gonzalez et al., 2015; Pennuto & Rupprecht, 2016; Tierney, Kasurak, Zielinski, & Higgs, 2011). The resulting relationship between body size and swimming speed that we applied here rests mainly on data presented in Tierney et al. (2011) (Figure 4). Assuming that the slope of the relationship between fish size and swimming speed in round goby is similar to other benthic fish, we extrapolated the Ucrit for fish larger or smaller than the sizes used in Tierney et al. (2011). Swimming performance and endurance taken from Tierney et al. (2011) were also recorded from experiments at temperatures ranging from 20 to 22°C (Tierney et al., 2011) and 17 to 20°C (Hoover et al., 2003). As such, experimental temperatures were comparable to summer temperatures at the time of flow measurements (20°C) and close to average summer temperatures in the studied section of the River Rhine (19°C) (see Section “2” Step 2 for more details). Hoover et al. (2003) specifically addressed the endurance of the round goby under experimental conditions. They provide a formula for the relationship between endurance time and flow velocity and size of an individual (sizes 9.1–15.4 cm).

### 3.3 Step 3. Analysis of fish personality traits, setting thresholds, and modeling the probability of ascent

An exemplary calculation of the decision matrix for one individual reads as follows: The size of the individual of 12.2 cm determines the swimming speed (following own calculations see above). This speed is higher than all measured bottom flow velocities at the 11 sampling sites (0.61 m/s >than max observed flow velocity 0.5 m/s). Thus, the first decision = 1. The size of the individual also determines the endurance in minutes sustained Ucrit swimming (following own calculations and Hoover et al.'s (2003) formula: _Log10_ Endurance = −0.027 [flow velocity in cm/s] + 0.007 [total fish length in cm] + 0.516). This endurance time measured as seconds with Ucrit as ground speed revealed a distance larger than the maximum distance between two pools in the bypass (13 m > 4.54 m). Thus, the second decision = 1. The individual's boldness index is higher than the mean for individuals sampled at the invasion front (1.71 > 1.13). The activity index is also higher than average (1.76 > 1.17), but not the asociability index (0.47 < 0.95). As we allow compensatory relationships between the three traits (see Section “3” for details), the third decision = 1. The overall decision = 1, which means that this individual ascended.

Calculating these steps of the decision matrix for all individuals revealed that approx. 18% of all individuals (9 of 50) successfully made an ascent. The logit regression model could significantly predict the success of ascent based on the input variables (2 × logLikelihood = 33.896, intercept = 55.108, χ^2^ = 21.212, *df* = 4, *p* < .001) and assigned 78% of all cases correctly. AIC and chi‐square likelihood ratio scores showed that of the four empirically measured parameters (length, boldness, asociability, activity), length and boldness were the most influential predictors for the modeled probability of ascent (Table 2).

**Table 1:** Length, boldness, and asociability significantly correlate with success. Spearman rank correlation coefficients between the success of ascent (0 or 1 as determined by the decision matrix) and the input variables. Significant correlation and contributions of the variables to the model at the *p* < .05 ‐level are marked in red

	Length	Asociability	Boldness	Activity	Success
Length	–	0.062	−0.066	−0.316	0.665
Asociability		–	−0.093	0.261	0.414
Boldness			–	0.366	0.464
Activity					0.265
Success					–

To explore linear correlations between success and failure and the predictor variables, we computed nonparametric rank correlations test (Spearman's *r*) and found that length, boldness, and asociability were significantly correlated with the success of the ascent. The intracorrelations of behavioral traits revealed that larger individuals tended to have lower activity and that bolder individuals were more active. Body length and boldness were not correlated (Table 1).

**Figure 5:**
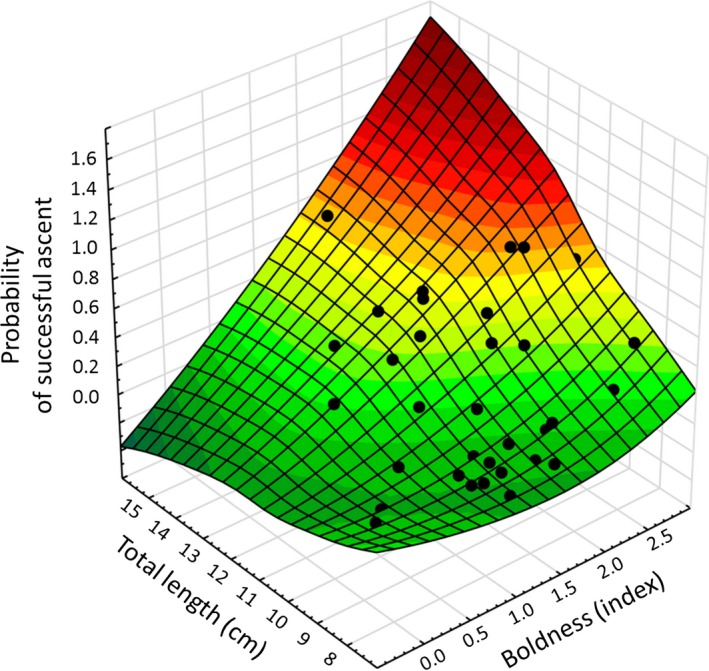
Larger and bolder individuals have a higher success in ascending the bypass. 3D plot of the model output exploring the relationship between boldness and length and the probability of success. The fitting is a surface generated by weighted least squares of the model fit. The fitting shows that whereas length and boldness are not correlated, both positively correlate with probability of successful ascent. The black dots indicate individuals with individual probabilities of success between 0 and 1 (100%). Please note that, for illustration purposes, the fitting (but not the actual values) exceeds the observed logical data limits of 0 and 1

The Hosmer–Lemeshow goodness‐of‐fit test revealed a χ^2^ = 3.056, with a clearly insignificant *p*‐value = .88, which indicates that there is no overinflation and that the model has a good fit of expected and modeled outcomes (Hill & Lewicki, 2006). The probability of success increased both with increasing boldness and increasing size (Figure 5; Table 2).

**Table 2:** Chi‐square values and AIC suggest that length and boldness are major predictors of success. Model building results of logit‐link function with binomial distribution for modeled probability of success

Variable 1	Variable 2	Variable 3	Variable 4	*df*	AIC	χ^2^	*p*‐Value
Length	Bold	Asoc	Act	4	43.896	21.212	.000
Length	Bold	Asoc		3	43.208	19.900	.000
Length	Bold	Act		3	46.887	16.221	.001
Length	Asoc	Act		3	47.393	15.715	.001
Bold	Asoc	Act		3	55.807	7.301	.063
Length	Bold			2	46.198	14.910	.001
Length	Act			2	48.401	12.707	.002
Length	Asoc			2	49.225	11.883	.003
Bold	Asoc			2	54.274	6.834	.033
Bold	Act			2	56.501	4.607	.100
Asoc	Act			2	59.243	1.865	.394
Length				1	50.197	8.911	.003
Bold				1	54.512	4.596	.032
Asoc				1	57.342	1.766	.184
Act				1	58.683	0.425	.515

Act, activity; Asoc, asociability; Bold, boldness.

## 4 Discussion

Here, we showed how empirical data on animal personality traits can be used to define thresholds in modeling dispersal across a migration barrier. Methodologically, this approach advances our ability to incorporate animal personalities into dispersal modeling. Conceptually, this approach advances and bridges disciplines by applying empirical data from behavioral ecology into the context of predictive modeling for ecological risk assessment.

### 4.1 Step 1. Flow measurements in the barrier

The flow velocity measurements provided a realistic empirical benchmark against which we could compare the estimated swimming speeds. Recent research in the emerging field of ecohydraulics has shown that structures such as stones can allow fish to ascend areas of flow which might be higher than their Ucrit (Maddock, Harby, Kemp, & Wood, 2013). Also for round goby, it has been shown that bottom substrate roughness can increase the ability to hold position against the flow (Hoover et al., 2003; Pennuto & Rupprecht, 2016). So we are confident that our measurements rather over‐ than underestimate the velocities that actually have to be overcome. Not least because measurements have to be viewed in light of the ecology of round goby, which is a fish that lacks a swim bladder and, as a benthic dwelling species, predominantly swims close to the bottom.

### 4.2 Step 2. Analysis of size‐dependent swimming speed and endurance time

The data we compiled for swimming speed in round goby and other benthic fish concur with the notion that the relationship between body size and swimming speed tends to be positive and linear in fish (Hoover et al., 2003; Pavlov, 1989; Tierney et al., 2011). A linear relationship is rarely a perfect description of nature, but the available data suggest that swimming speed and fish length are indeed quasi‐linearly correlated for most swimming styles, such as sustained, prolonged, and burst swims (Kieffer, 2000). Several studies have also demonstrated that round goby morphology and especially fin size and aspect ratios scale isometrically with size in adult fish (Lavrincikova & Kovac, 2007; Pennuto & Rupprecht, 2016). This suggests that morphological traits, which can influence swimming speed such as caudal fin aspect ratio (Radinger & Wolter, 2014), do not allometrically change across the size ranges of fish we studied here. Based on this background information, it is a logical result that length was a significant predictor in our model: We assumed a linear relationship between size and swimming speed and the latter would ultimately decide in the matrix whether an individual can overcome the highest measured velocity or not.

### 4.3 Step 3. Analysis of fish personality traits, setting thresholds, and modeling the probability of ascent

The results for boldness and asociability indicate that a specific personality trait can serve as a proxy for the intention to ascend and be incorporated into a dispersal. In our decision matrix, we were blind toward which above‐average trait of behavior eventually decides over success or failure. We did not a priori weigh any of the traits more than the other. In that respect, our model provides a probabilistic expression of the influence of relevant behavioral parameters for a probability of ascent. In fish, there is clear evidence that bold and more exploratory individuals can lead a shoal of fish into novel environments (Schöpf Rehage & Sih, 2004; Thorlacius et al., 2015). In roach (*Rutilus rutilus*), boldness has been clearly linked to the propensity to migrate from lakes into adjacent streams (Chapman et al., 2011). When killifish (*Rivulus hartii*) were assayed for boldness in tanks and then released, the level of individual boldness predicted the distance moved in the field. Interestingly, this effect remained even after co‐founding effects such as size were accounted for (Fraser, Gilliam, Daley, Le, & Skalski, 2001). In round goby, bolder individuals were found to move farther distances in artificial flumes than shyer ones (Myles‐Gonzalez et al., 2015). Collectively, these empirical results are reflected in the models assumptions. This allows for a more comprehensive risk assessment which acknowledges that personality traits might facilitate not only dispersal through a landscape but also passage over a barrier.

### 4.4 Risk assessment with incomplete knowledge

Our approach here should not be mistaken for statistical analyses of migration. As, for example in migratory birds, such analyses are based on large quantities of empirical data on typically GPS‐tracked individuals Here, instead we built a model to incorporate personality measures of a spreading invasive species with the aim of making informed assessments of the actual risk of spread across a barrier. We based our model on in situ flow measurements, on empirical data from behavioral experiments, and on published length‐swimming speed relationships. Our model is aimed at a risk assessment of goby upstream dispersal in a situation where observable evidence of how fish actually perform in the field is unobtainable. We therefore had to set the binary decision of success or failure on a priori thresholds which, although based on own measurements or literature data, could not be based on field observations. This makes the model naturally artificial, but it also serves its purpose of providing an assessment tool of how a barrier actually affects nonmigratory fish which do not have a “constant intention” to ascend. By definition, any risk assessment must happen before the actual risk started to affect an ecosystem at which point it becomes an empirically testable reality. Until then, researchers and decision makers need tools that produce scenarios of what could happen if the risk unfolds (Hirsch, N'Guyen, Adrian‐Kalchhauser, & Burkhardt‐Holm, 2016; N'Guyen, Hirsch, Adrian‐Kalchhauser, & Burkhardt‐Holm, 2016). Our study aimed to create such a scenario by incorporating animal personality as a previously underappreciated factor into the risk assessment of a spreading invasive species.

### 4.5 Improving existing modeling frameworks by including personality data and acknowledging dispersal barriers dispersal

Our model's purpose of incorporating individual personalities into the risk assessment of a spreading invasive population can be further advanced for a specific case by more elaborate modeling. For example, parameterized round goby population models from the Great Lakes provide estimates of the propagule pressure needed for an invasive round goby population to establish (Taraborelli, Fox, Johnson, & Schaner, 2010). Such estimates of how many individuals are needed to found a new population can also be combined with genetic data on the percentage of migrants which can be inferred by population genetics of spreading round goby populations (Bronnenhuber, Dufour, Higgs, & Heath, 2011). Our model could serve as an addition to these existing approaches by incorporating individual personalities which can affect not only dispersal but also the success of population establishment. Models of bird introductions have shown that the prediction of establishment success is improved if behavioral syndromes are incorporated into the models (Sol, Duncan, & Blackburn, 2005; Sol & Lefebvre, 2000). To our knowledge, this approach has not yet been taken for modeling dispersal across migration barriers. Traditionally, the assumptions for modeling dispersal seldom include barriers (Brownscombe, Masson, Beresford, & Fox, 2012). Even elaborate dispersal models of fish have not considered differences in the “intention” of individuals to overcome a barrier (Meixler, Bain, & Walter, 2009; Morales et al., 2010). Our approach might also allow for better risk assessment of invasion scenarios through an improved understanding of rates of natural spread because existing dispersal models can be extended to account for the frequently fragmented nature of aquatic ecosystems. A common approach to model fish dispersal more probabilistically assumes so‐called dispersal kernels (Radinger & Wolter, 2014). These kernels follow the logic that any population consists of a few mobile individuals that realize dispersal “at the front of a population” (Radinger & Wolter, 2014). The quantification and characterization of these dispersers, however, rarely rests on empirical field data (but see Fraser et al., 2001 for an exception) and is typically not adjusted for dispersal across barriers. Traditionally, a leptokurtic distribution is assumed: a few mobile individuals at the flat tails and a majority of resident individuals in the middle of a bell‐shaped curve (Fraser et al., 2001; Radinger & Wolter, 2014). Our data on personality traits, assayed at the level of a possible source population, showcase the potential to make more specific estimates of the amount of mobile individuals within a putative dispersal kernel. A more realistic estimate of the number of dispersers arriving in a new environment will allow for more robust risk assessments for population establishment beyond a barrier. Based on the theory of propagule pressure, an assessment of how many individuals will spread across a barrier can improve predictions of how likely population establishment in new environments above barriers will be (Drolet & Locke, 2016; Lockwood, Cassey, & Blackburn, 2005). This information is crucial for decision makers both in and outside academia, who increasingly need to balance the need for making barriers passable to native species and the opportunity of blocking invasive species’ dispersal (Frings et al., 2013; Hirsch et al., 2016).

### 4.6 Reliability and robustness of behavioral thresholds

The most critical steps in our model were the assumptions for the input variables and the definition of thresholds. The emerging field of ecohydraulics strongly advocates for more realistic models of fish swimming performance that acknowledge biotic and abiotic parameters (Maddock et al., 2013). Current publications emphasize the necessity for a detailed analysis of in situ movements before fitting more elaborate movement models (Gurarie et al., 2016). The indirect measure of swimming speed we applied as a function of size is clearly a simplification of more complex ecological relationships. More empirical work such as mark–recapture studies or telemetry studies in combination with swimming performance measures of recaptured individuals could give more insight into size‐dependent swimming performances within barriers (Hirsch, Burkhardt‐Holm, Töpfer, & Fischer, 2016; Johnson et al., 2012). This however, can only be carried out in already invaded systems. Defining plausible thresholds for risk assessment a priori is notoriously challenging (Copp et al., 2009) and in the case of invasive species decisions need to be made with incomplete knowledge (Leung et al., 2002). In this study, we based our model assumptions on incomplete knowledge of real outcomes and gleaned the behavioral determinants of successful ascent from own empirical data. The demonstrable differences in personality between individuals from invasion centers and invasion fronts suggest that our decision matrix included plausible a priori decisions for personality thresholds. However, one obvious question concerning the thresholds is whether a threshold derived from multiple geographically distant sampling sites and contexts can be applied to a specific case study. Previous research on animal personalities has specifically addressed the topic of context dependency. Interestingly, boldness was frequently found to be rather context‐independent in fish. For example, risk‐taking behavior in Gilthead seabream (*Sparus aurata*) is independent of nutritional status and hypoxia (Castanheira, Herrera, Costas, Conceição, & Martins, 2013). However, the consistency of personality traits such as asociability and activity was frequently found to be affected by social contexts and nutritional status (Cote et al., 2011). Although we did not attempt to identify major differences statistically, it is obvious that, across all our data, the differences between individuals from invasion centers and invasion fronts were highest for boldness. By definition, asociability and activity may also be influenced more by social context and external factors such as temperature than one would expect of boldness. The context independence of boldness might make it a strong predictor of dispersal in empirical work and also a potentially workable proxy for quantifying the intention of ascent in an effective way. How robust asociability and activity are as model parameters across different contexts remains to be studied. At any case, our study's data can provide the basis for further numerical modeling and empirical research.

### 4.7 Appreciating animal personalities in the Anthropocene

Recent conceptual work has put more emphasis on integrating individual personalities into research on animal movements (Canestrelli et al., 2016). For example, intraspecific personality differences can create a subsample of bold “colonizer‐phenotypes” establishing a population beyond migration barriers (Hale, Morrongiello, & Swearer, 2016). In‐stream barriers in rivers could thus selectively remove “colonizer‐phenotypes” from a downstream population by acting as a filter for specific personalities (Hale et al., 2016). Such a selection of phenotypes determines the population's phenotypic variability upon which natural selection can act and thus affects the potential for genetic change (Beechie et al., 2013; Hirsch, Eckmann, Oppelt, & Behrmann‐Godel, 2013). In the case of invasive species, the effect of barriers selecting out specific phenotypes has further implications for management options. Current research poses the question of whether the removal of colonizer‐phenotypes selected by a barrier could affect population persistence (Hale et al., 2016). The planning of management efforts for invasive populations could therefore benefit from acknowledging that removal targets are a nonrandom selection of the population. For example, bioacoustics traps are currently developed as management tools for round goby which can selectively target specific reproductive phenotypes (Isabella‐Valenzi & Higgs, 2016). Such novel tools could potentially be tailored to remove “colonizer‐phenotypes.” The filtering function of barriers also adds an important dimension to the topic of creating barriers to prevent species invasions. Today, so‐called prescribed disconnections to protect pristine habitats are a widely discussed tool for the prevention of species invasions (Frings et al., 2013; Hermoso et al., 2015). Research on the effectiveness of such management tools should appreciate the importance of animal personality in determining the propensity to overcome prescribed disconnections such as in‐stream barriers (Frings et al., 2013; McLaughlin et al., 2013). In the Anthropocene, where most ecosystems are highly fragmented, the approach of including personality traits into dispersal modeling across barriers holds promise to advance both conservation and invasive species management planning.

## Conflict of Interest

None declared.
