# Supplementary material for: An approach to incorporate individual personality in modeling fish dispersal across in‐stream barriers
Source: Ecol Evol. 2016 Dec 23;7(2):720–32. doi: 10.1002/ece3.2629 (PMC5243775; doi:10.1002/ece3.2629)
Supplement: Supplementary file 1 [file ECE3-7-720-s001.docx]

**Appendices for**

AN APPROACH TO INCORPORATE INDIVIDUAL PERSONALITY IN MODELLING FISH DISPERSAL ACROSS IN-STREAM BARRIERS

P. E. HIRSCH * M. THORLACIUS, T. BRODIN, P. BURKHARDT-HOLM

*philipp.hirsch@unibas.ch

**Appendix S1**

Detailed profiles of flow velocity measurements in the bypass. In order of measurement site numbers as seen in Figure 1 C of the main manuscript. The two colours of the bars indicate measurements conducted in the centre of the bypass flow and near the shoreline of the bypass.

**
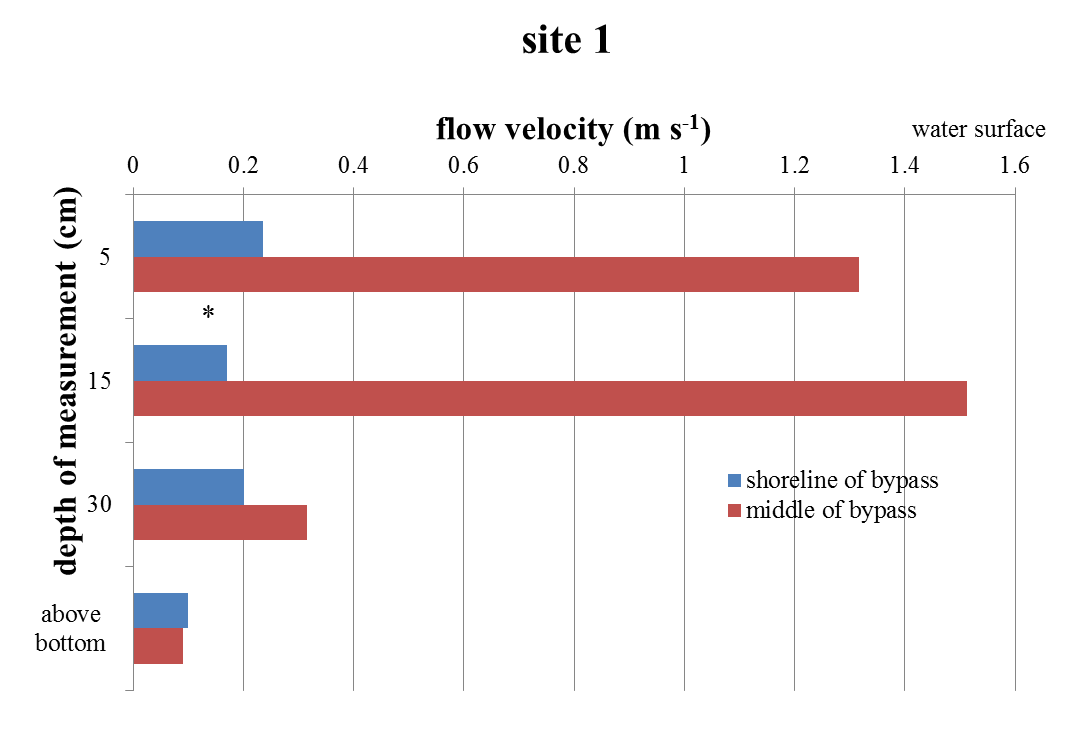
**

**
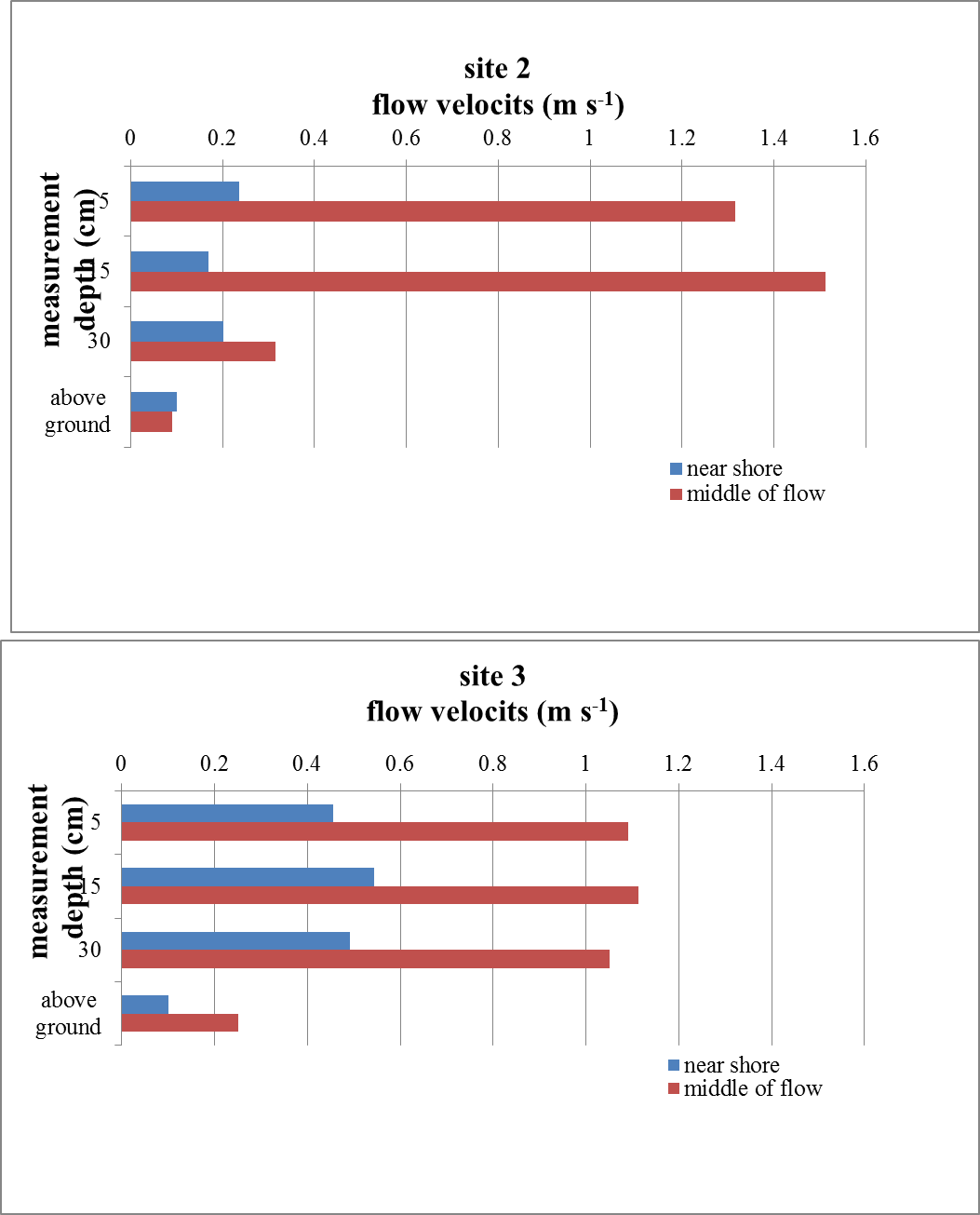
**

**
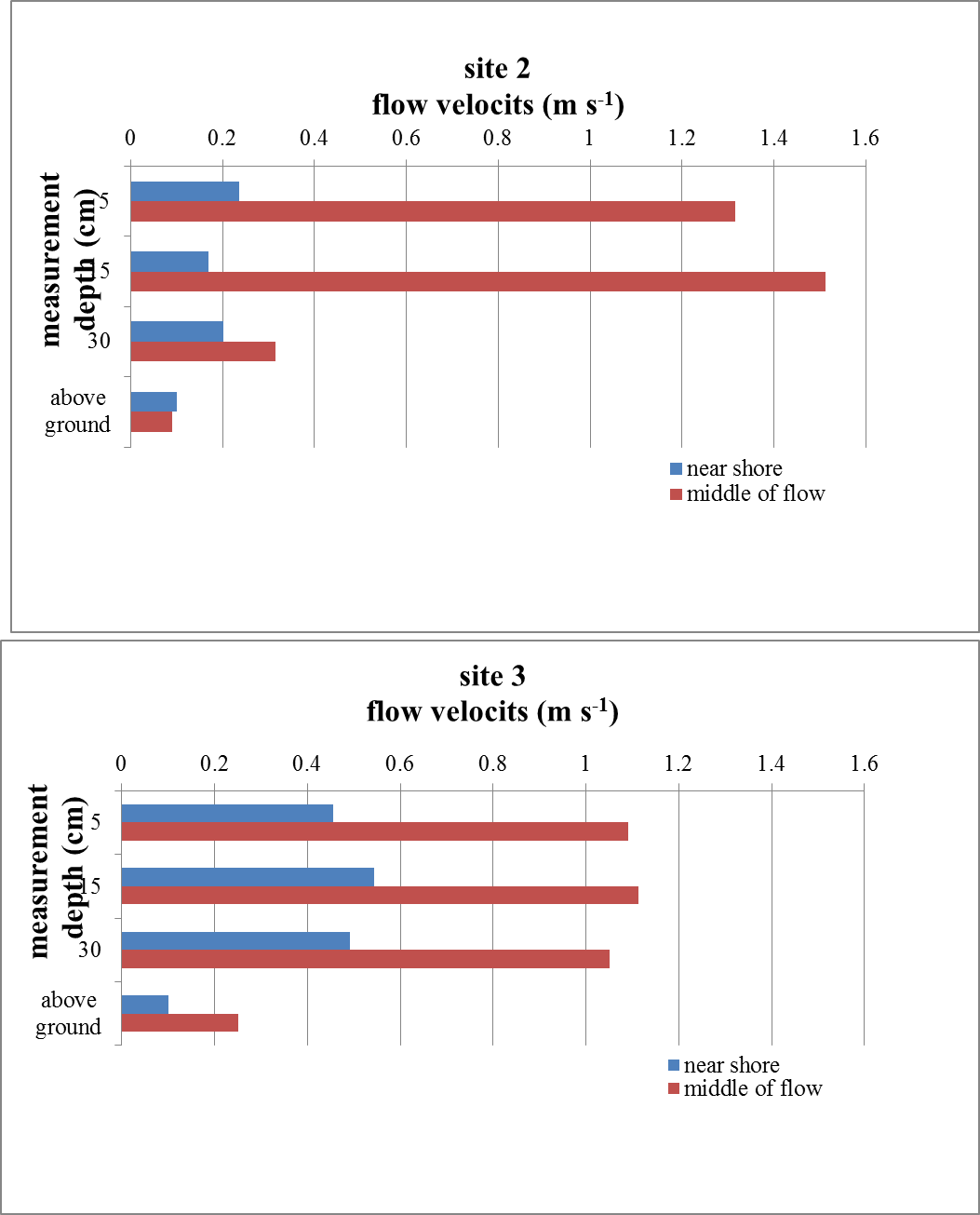
**

**
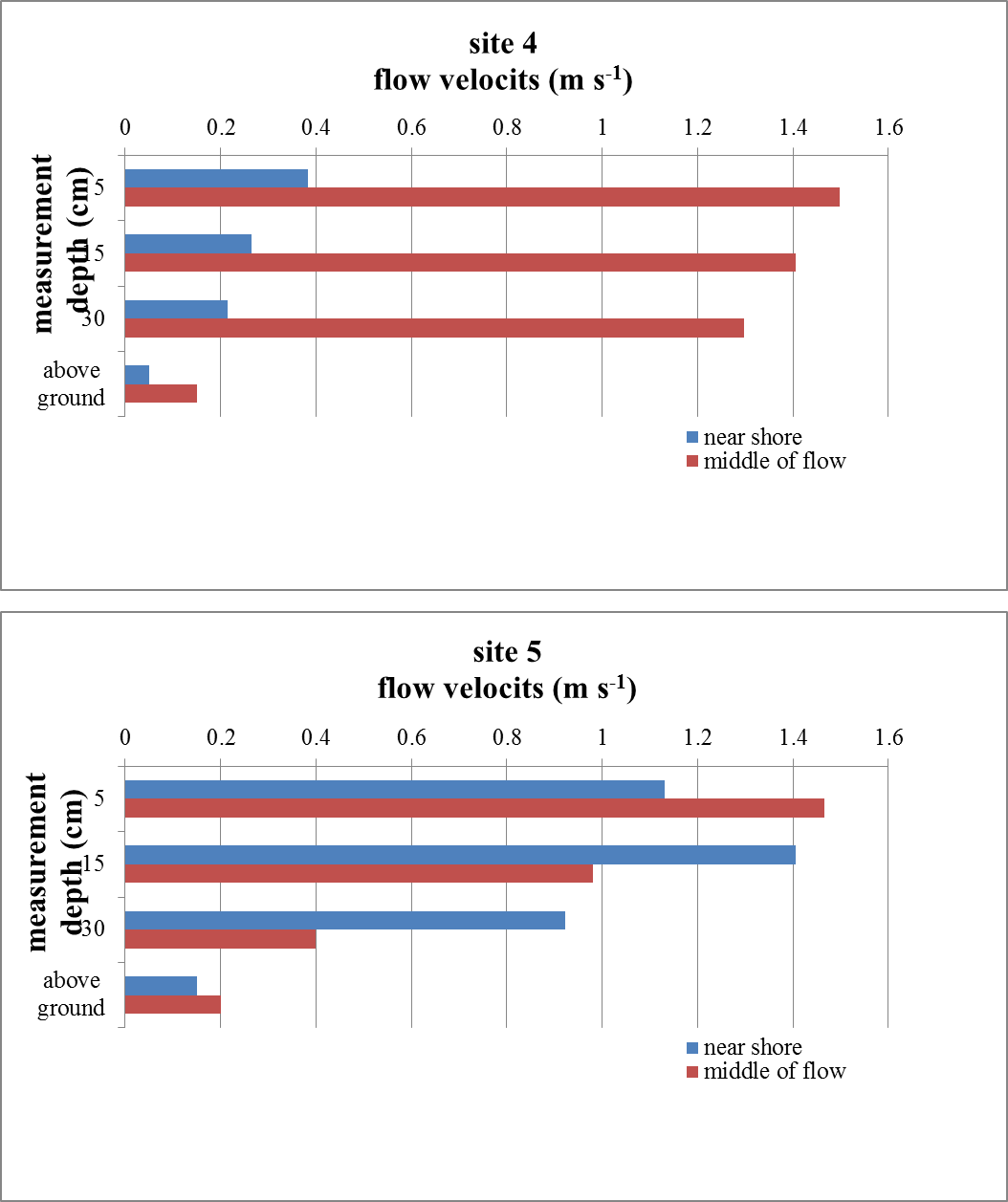

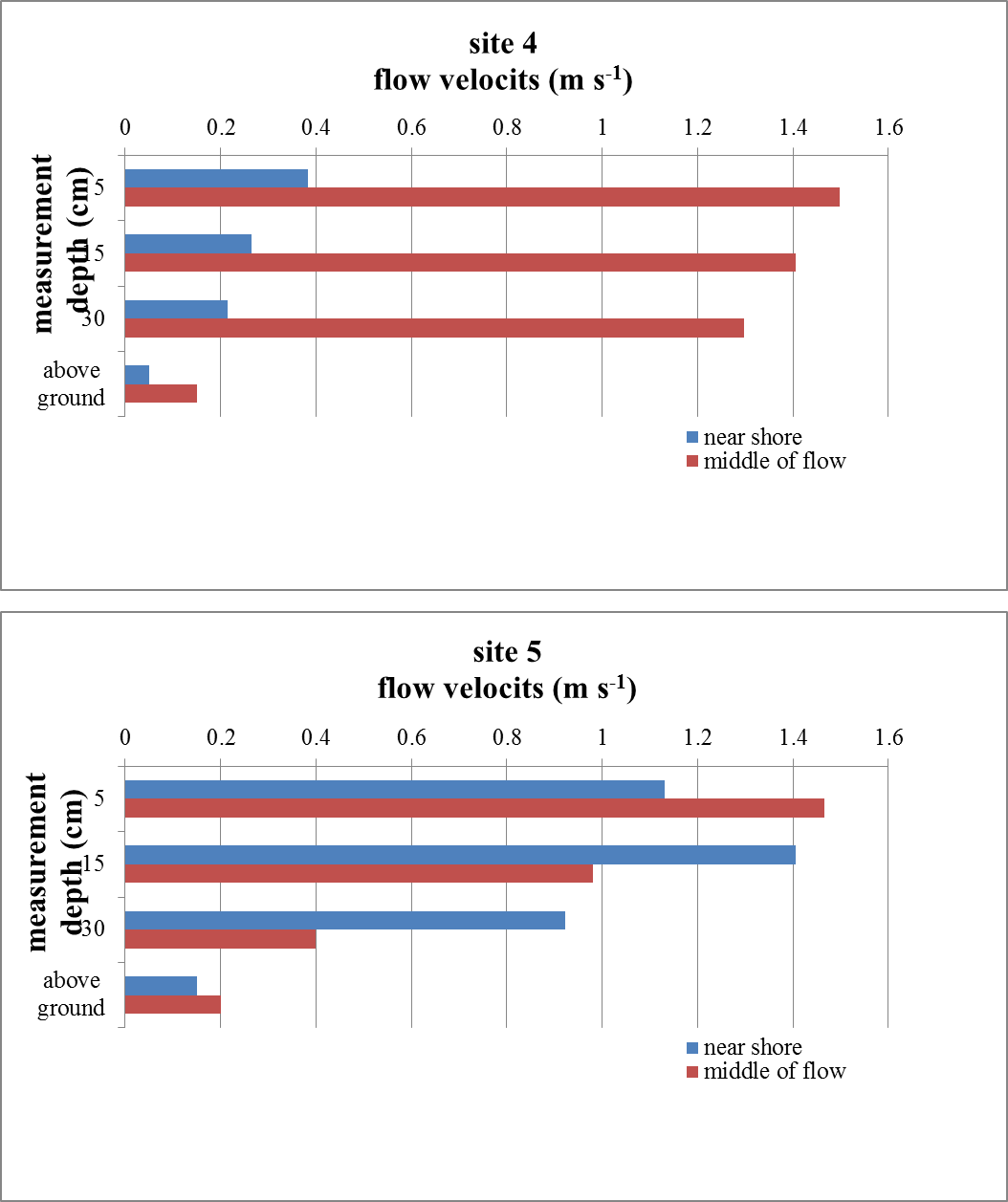
**

**
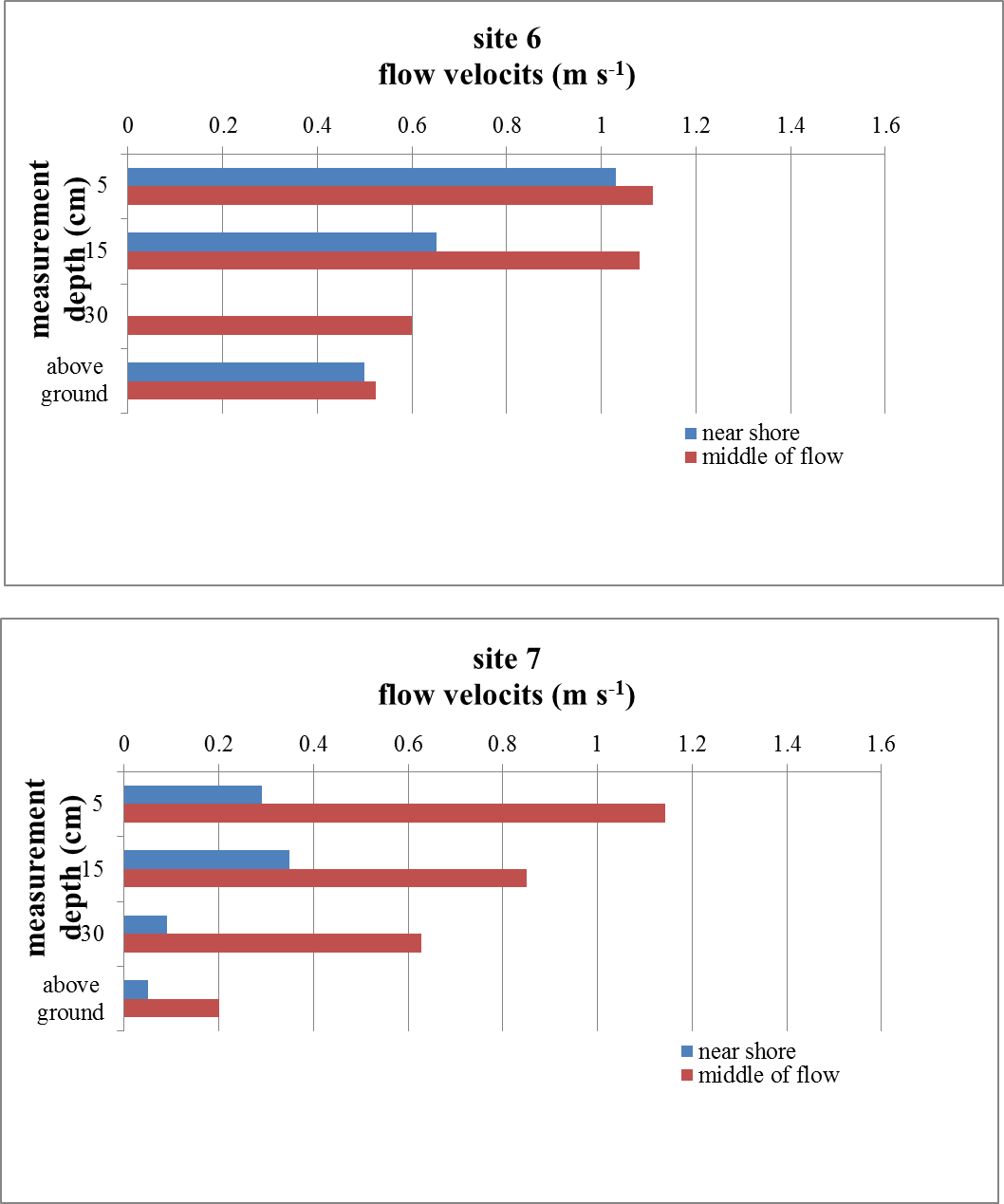
**

**
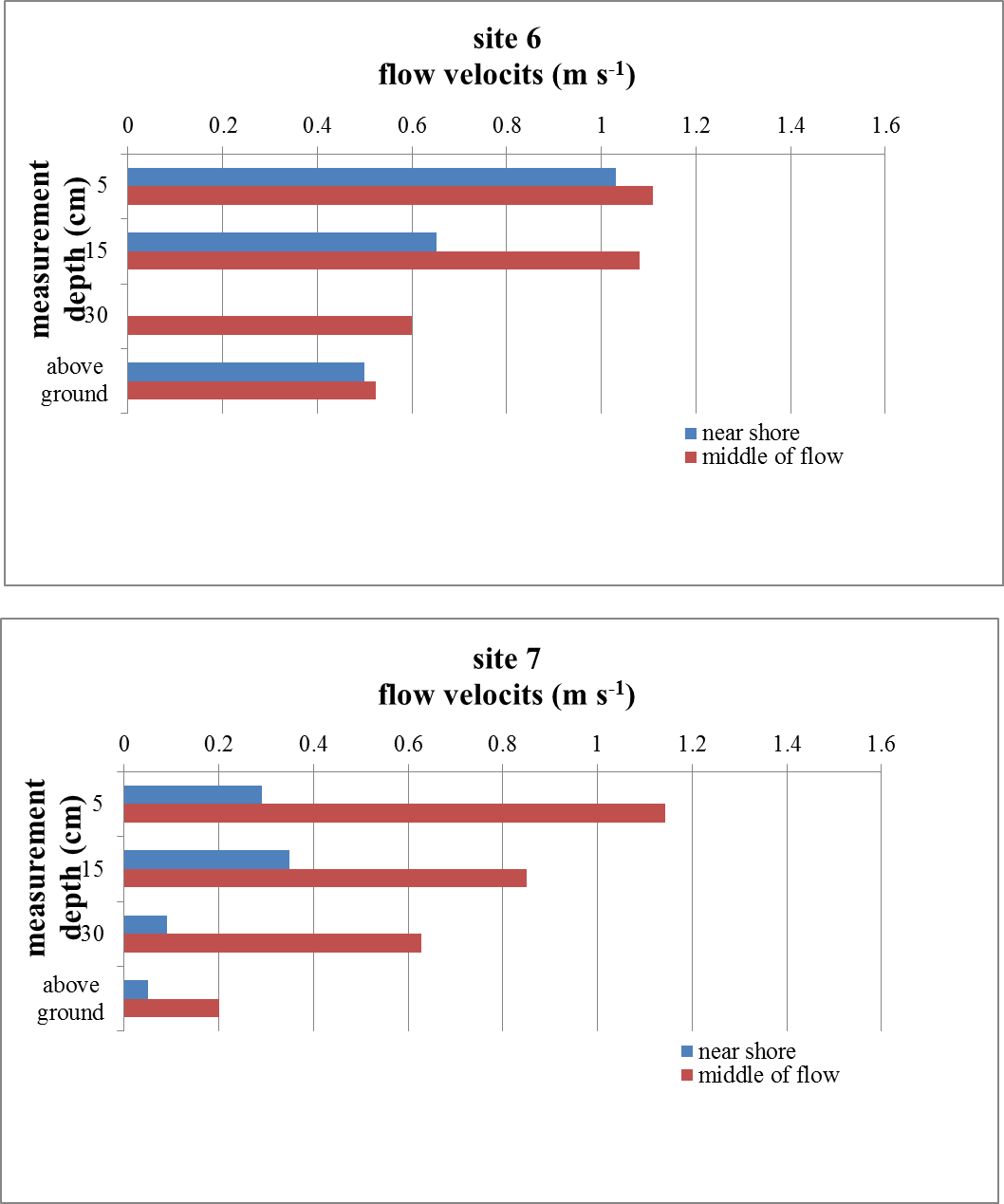
**

**
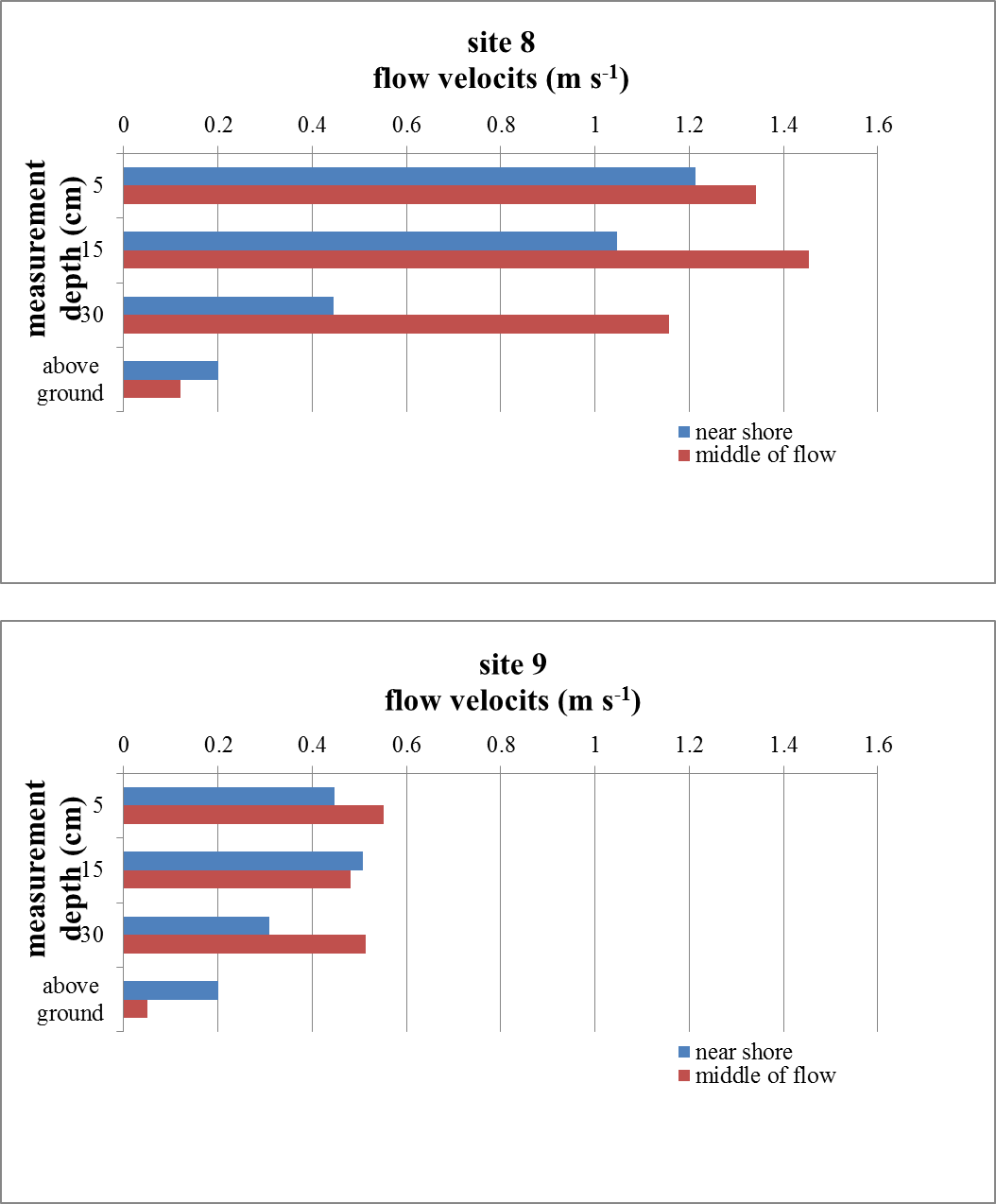
**

**
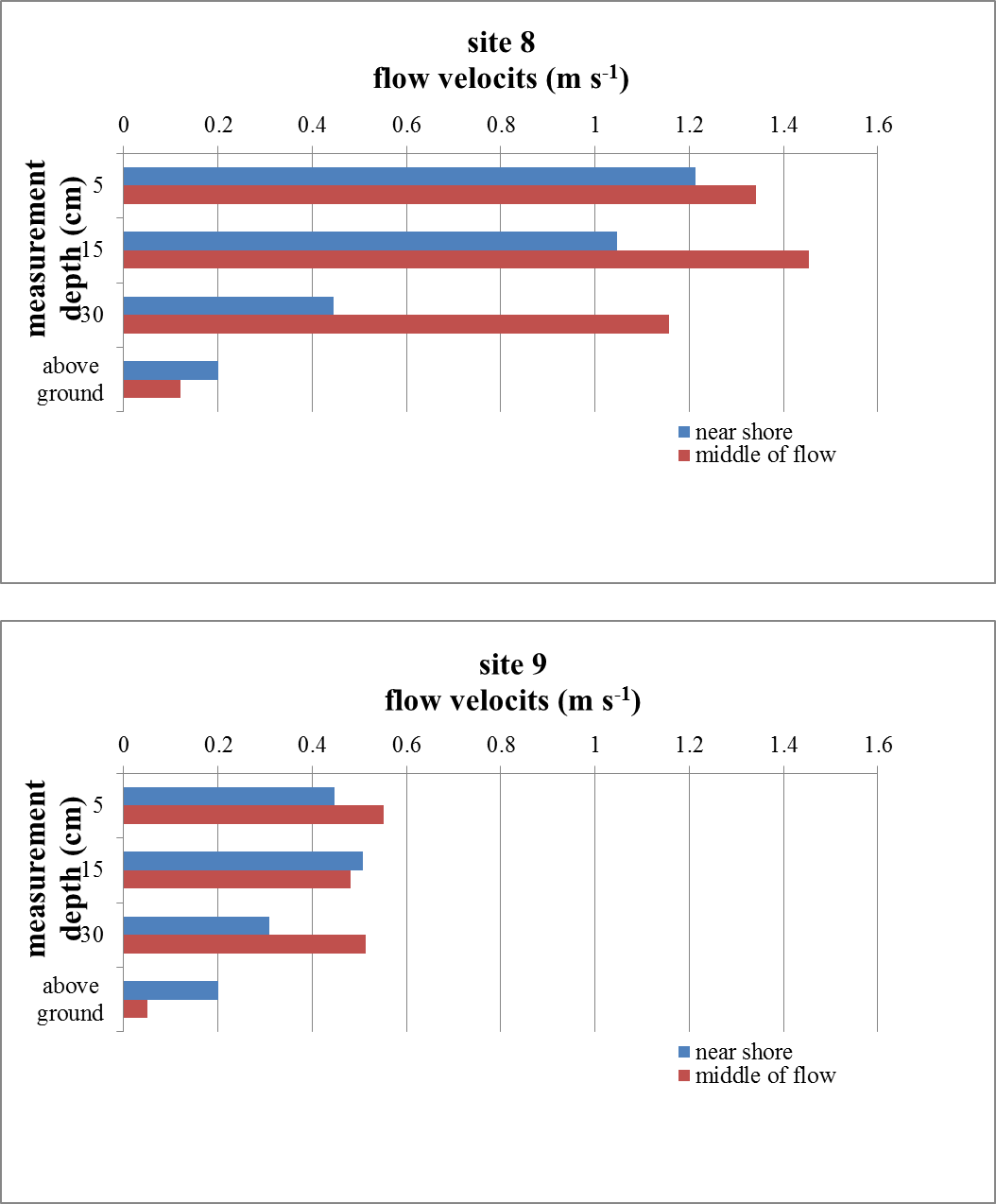
**

**
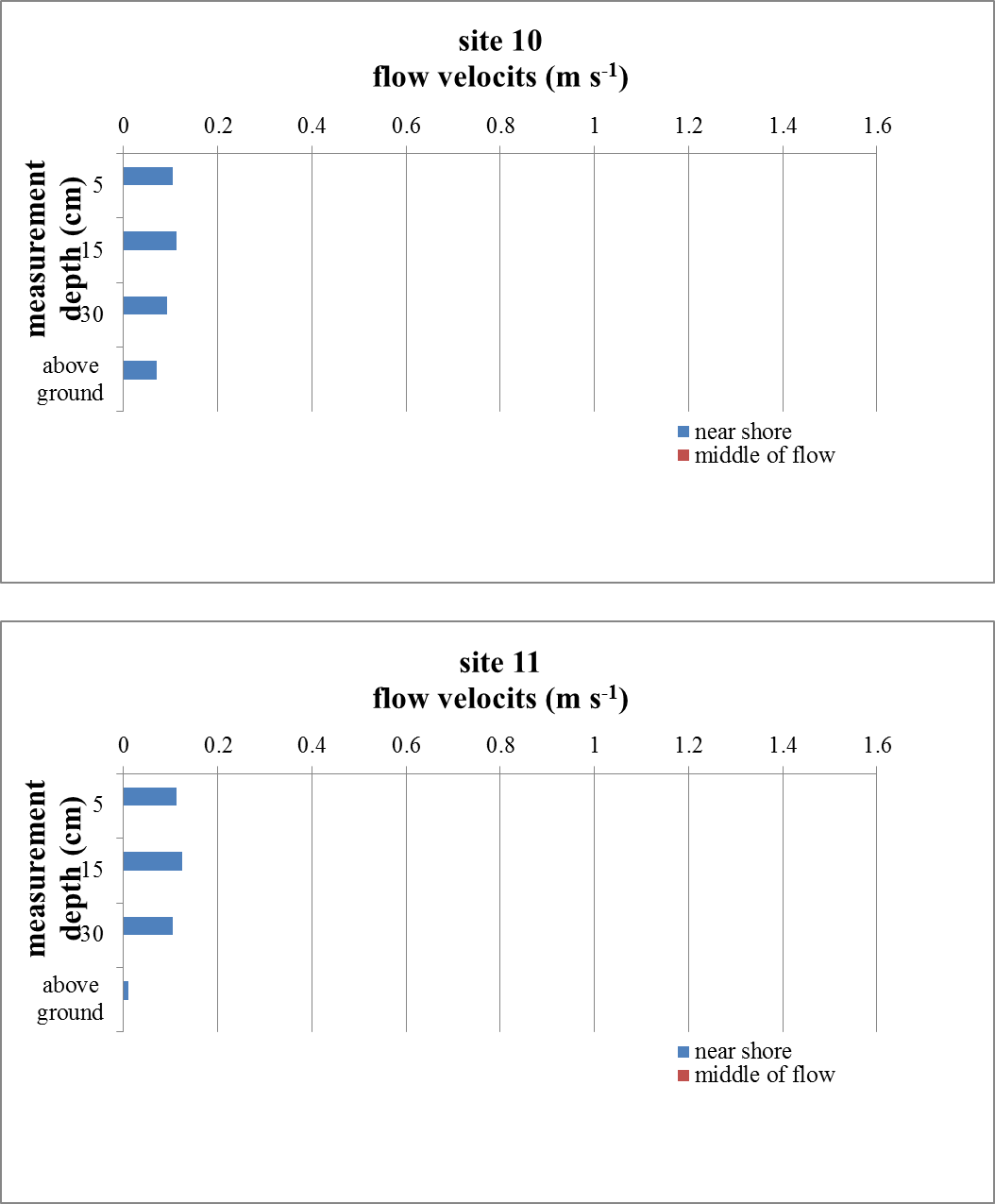

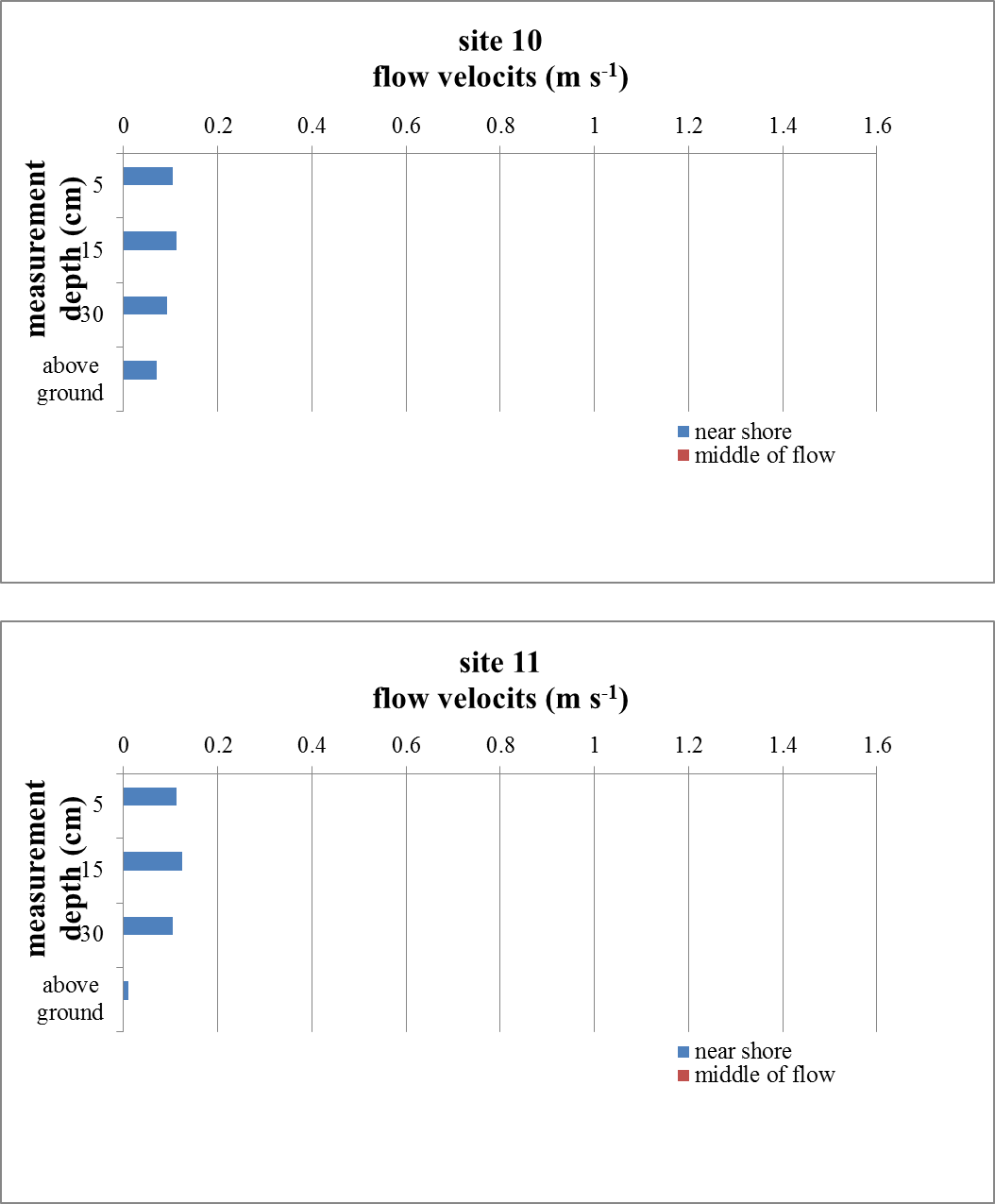
**

**Appendix S2**

Sampling in all locations was conducted using fyke-nets with mesh size 11-20 mm similar to the type applied for sampling the invasion front and possible source population in Basel, Switzerland (see main manuscript). The nets were left in the water for 24 hours on each catch site. Individuals were transported from their respective catch sites to the marine station (Umeå Marine Science Centre) where they were all kept separated by their origin in large basins (110x110x100 cm) as described in the main text with a light:dark cycle of 14:10 hours. Different from the individuals from the River Rhine in Basel, the tanks contained natural brackish water from the Bothnian Bay (0.4 % salinity) with a constant flow-through. All tanks contained one shelter per individual and fish were fed with pellets from Skretting Nutreco® three times per week. Marking with Pit-tags and acclimatization followed the same procedure as described in the main text. Body length varied across sampling sites of invasion front and centre but there was no consistent difference between front and centre in terms of size (ANOVA with catch site as fixed factor: df=1, MS=613.74, F=1.57, p=0.29; Figure S2.1). For details on the catch sites please refer to the main text.


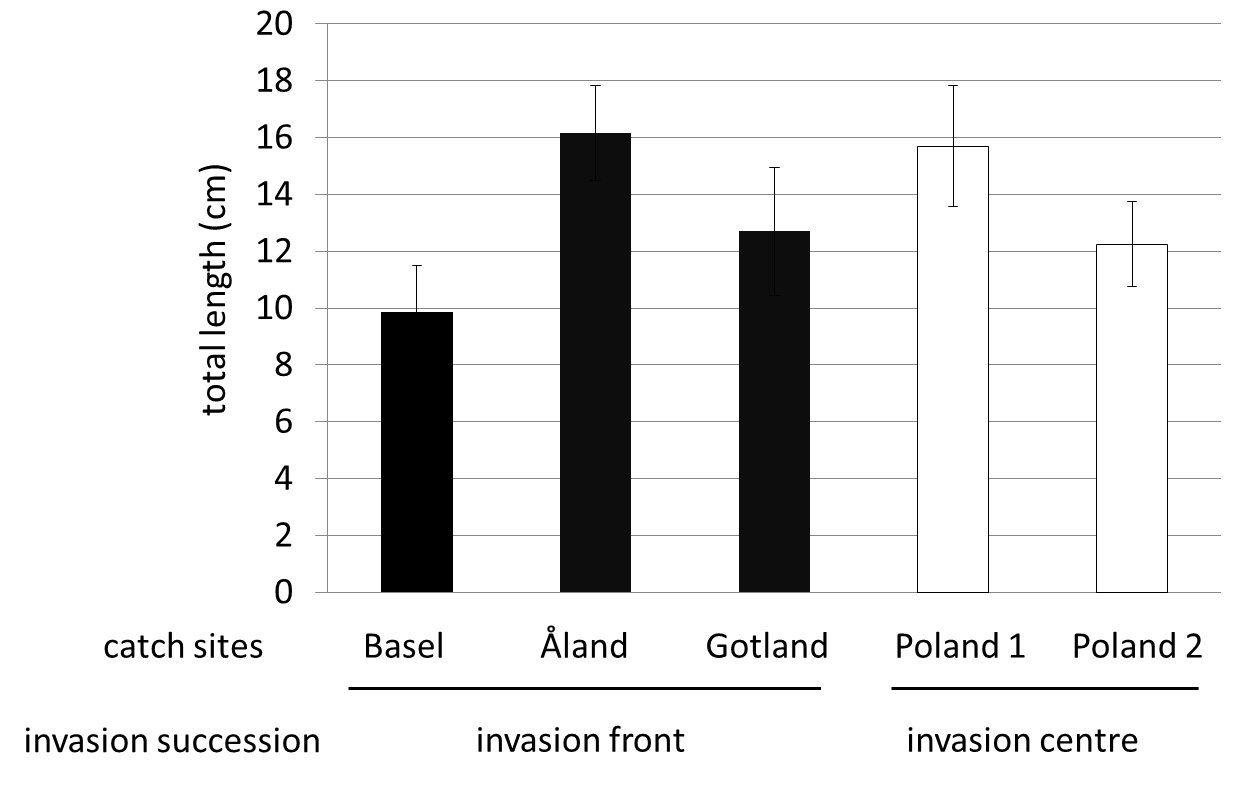


Figure S2.1. Body size in total length of fish from the different catch sites for invasion fronts and invasion centres. See main text for detailed geographical information.

**Appendix S3**

In all studies boldness was measured as latency to recover after a risky encounter in the form of a novel object in the Baltic populations and an imitated bird strike in the population from Basel. All individuals were essayed twice to be able to test for repeatability. To be able to combine activity data, all activity data was converted to percentage of the maximum ((n/300) x 100 for Baltic and (n/600) x 100 for Basel) to account for different times of trials. In both studies, individuals that did not move in the first 30 minutes were excluded from the analysis because sufficient data could not be gathered.

**Appendix S4**

To ensure that an arithmetic mean represents an objective cut-off we explored the distribution of the behavioural data statistically. We found that neither trait were strongly skewed or had extreme kurtosis which would be defined as values larger than ± 3 ([Kline 2005](#_ENREF_21)). Because kurtosis above or close to zero means that the distribution is leptokurtic (meaning fatter tails and lesser risk of extreme outcomes) we deemed the arithmetic mean of each trait a reasonable threshold from a mathematical point of view.

**References**

Kline, R.B., 2005. Principle and practice of structural equation modeling, New York: Guilford

Figure S4.1. Frequency histogram of the three behavioural traits activitity (act, blue), asociability (asoc, orange), and boldness (bold, green). A Lowess fit indicates a near-gaussian distribution in the data. All data are presented as dimensionless indices. See main text for more details.

**Appendix S5**

List of species with references used to confirm that swimming speed and body size have a quasi-linear positive relationship. Values were taken directly as given in the paper but if necessary data were extracted from figures using a graph digitizer (graph-data extractor).

| **Graphical depiction as in figure** | **Species (authority)** | **Reference** |
| --- | --- | --- |
| 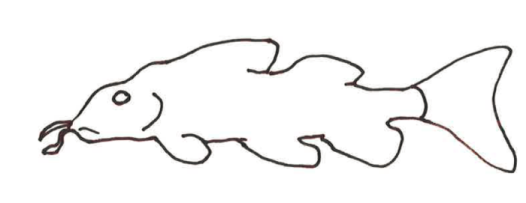 | *Leporinus reinhardti* (Liitken, 1875) | de Andrade e Santos, H., de Faria Viana, E.M., Pompeu, P.S., Martinez, C.B., 2012. Optimal swim speeds by respirometer: an analysis of three neotropical species. Neotropical Ichthyology 10, 805-811. |
| 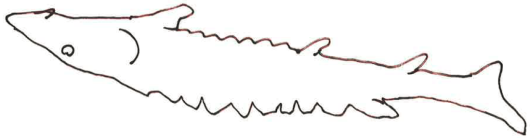 | *Acipenser medirostris* (Hilgendorf, 1892) | Verhille, C.E., Poletto, J.B., Cocherell, D.E., DeCourten, B., Baird, S., Cech, J.J., Fangue, N.A., 2014. Larval green and white sturgeon swimming performance in relation to water-diversion flows. Conservation Physiology 2. |
| 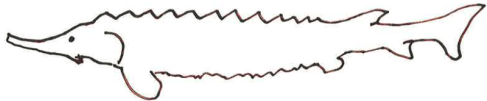 | *Acipenser transmontanus* (Richardson, 1836) | Verhille, C.E., Poletto, J.B., Cocherell, D.E., DeCourten, B., Baird, S., Cech, J.J., Fangue, N.A., 2014. Larval green and white sturgeon swimming performance in relation to water-diversion flows. Conservation Physiology 2. |
| 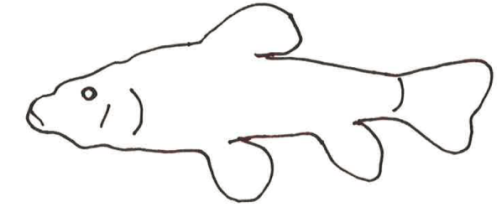 | *Erimyzon sucetta*  (Lacepède, 1803) | Peake, S.J., 2008. Swimming performance and behaviour of fish species endemic to Newfoundland and Labrador: A literature review for the purpose of establishing design and water velocity criteria for fishways and culverts. Canadian Manuscript Report of Fisheries and Aquatic Sciences 2843, i-v, 1-52. |
| 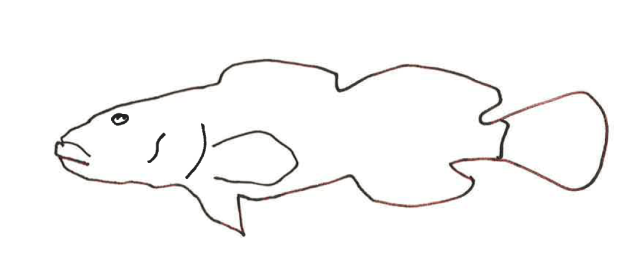 | *Cottus gobio*  (Linnaeus, 1758) | Pavlov, D.S., 1989. Structures assisting the migrations of non-salmonid fish: USSR., In FAO Fisheries Technical Paper. p. 97. FAO., Rome. |
|  | *Cottus bairdi*  (Girard, 1850) | Peake, S.J., 2008. Swimming performance and behaviour of fish species endemic to Newfoundland and Labrador: A literature review for the purpose of establishing design and water velocity criteria for fishways and culverts. Canadian Manuscript Report of Fisheries and Aquatic Sciences 2843, i-v, 1-52. |
